# Migratory blackpoll warblers (*Setophaga striata*) make regional-scale movements that are not oriented toward their migratory goal during fall

**DOI:** 10.1186/s40462-017-0106-0

**Published:** 2017-07-03

**Authors:** J. Morgan Brown, Philip D. Taylor

**Affiliations:** Department of Biology, Acadia University, Wolfville, NS B4P 2R6 Canada

**Keywords:** Migration, Stopover, Movement, Blackpoll Warbler, Automated Radio Telemetry, Songbirds

## Abstract

**Background:**

Regional scale movement patterns of songbirds are poorly known largely due to difficulties tracking small organisms at broad scales. Using an array of over 100 automated radio telemetry towers, we followed Blackpoll Warblers (*Setophaga striata*) during fall migration in the Gulf of Maine region, and assessed how their regional scale movement pathways varied with age, distance to natal origin, and capture date.

**Results:**

Many individuals had movement paths that were not oriented towards their migratory goal (‘indirect movement patterns’), regardless of age, distance to natal origin, or time of season. The probability of moving in indirect patterns, and the total tracking duration, decreased with capture date. The extent of indirect movement patterns varied considerably between individuals. Excluding direct flight patterns consistent with traditional migratory movements, adults tended to make more flights and moved in more tortuous patterns than hatch-years. Adults and individuals from more westerly natal origins were more likely to move south-west through time.

**Conclusions:**

A greater proportion of individuals made movements that were not oriented towards the migratory than expected. A decrease in tracking duration with capture date indicates that individuals prioritize time as the season progresses. The shorter, indirect movement patterns may be a more complete representation of ‘reverse migration’ at a barrier or ‘landscape-scale stopovers movements’. The longer distances travelled are inconsistent with expected behaviour, even in front of a barrier. The extent of movement we observed indirectly suggests that flight is not as costly to individuals in a migratory state as is commonly assumed. Since adults were observed to move more than hatch-years, we suggest that the indirect movement patterns we observed are not accidental, and may provide some advantage to the individuals that undertake them.

**Electronic supplementary material:**

The online version of this article (doi:10.1186/s40462-017-0106-0) contains supplementary material, which is available to authorized users.

## Background

Landscapes are organized at multiple scales, within which animals must make hierarchical decisions regarding movements [1–4]. At the broadest (continental) scale, migration is a seasonal, lengthy movement, where an individual follows innate directions towards a specific location [5]. The study of passerine migration at broad scales has a long history [6, 7] and the overall migratory journey is well described for many bird species. Patterns of movement at regional scales, however, are poorly understood, largely due to the lack of tracking devices small enough for most passerines to carry, while still providing sufficient spatial and temporal accuracy [8].

At regional scales, individuals make decisions about the specific movement paths they take through landscapes. An individual’s realized movement path is considered a function of its internal state, motion capacity, navigational capacity and its interactions with the external environment [9]. Empirical studies of path choice are particularly under-represented in studies of movement ecology, not just in birds, but across all taxa [10]. Studying individual path choice during periods of high mobility has been identified as one of the main missing elements for the effective conservation of migratory birds [11].

In migratory passerines, a bird in its first year of life (“hatch-year”) will be embarking on its first migration during fall, whereas adult individuals have successfully completed at least two migrations (one fall, and one spring). This difference in migratory experience has been demonstrated to effect an individual’s efficiency (in terms of time and energy use) during migration. For example, adult Savanna Sparrows (*Passerculus sandwichensis*) are more selective of the nights on which they depart on a migratory flight and are better at obtaining energy savings with the assistance of tailwinds [12], compared to hatch-years. In some species, adults are also more efficient flyers [13, 14]. Compared to adults, hatch-years migrate at a slower pace [15–18] and spend more time at refuelling sites [19–23]. Differences between adult and hatch-year individuals may decrease through time as hatch-years gain more experience in migratory behaviour [13, 24, 25]. In addition, adult birds migrate using a ‘navigational map’ learned during their first migrations, allowing them to correct for displacements by using alternate migratory paths [26–29]. Hatch-year individuals, however, likely rely on dead-reckoning, limiting them to genetically-programmed migration routes and limiting their ability to correct for displacement ([26] but see [30]). In summary, during fall migration hatch-year individuals experience both increased energy expenditure (and thus are more likely to be energy limited) and have reduced navigational capacities compared to adults. Age can therefore be used as an indicator of both the internal state and navigational capacity of an individual.

External factors such as ecological barriers, weather, and time of season can also influence path selection in migratory songbirds. A migratory barrier is an area, such as open water, that provides no suitable habitat for a migrant to stop. Barrier crossings can therefore be risky, because birds cannot land if they encounter inclement weather conditions or expend their energy reserves [31, 32]. Whether a barrier is perceived as risky can depend on age, with hatch-year individuals being more likely to avoid or shorten a barrier crossing [33, 34], and the barrier’s size (relative to the species’ migration route) [35]. Small songbirds can be significantly assisted (or impeded) by winds, so altering flight direction to increase tailwind can be beneficial, even if it results in a detour from the most direct migratory route [36]. Individuals migrating later in the season are under more time pressure, because winter weather conditions are more imminent and food resources become depleted [37, 38].

**Fig. 1:**
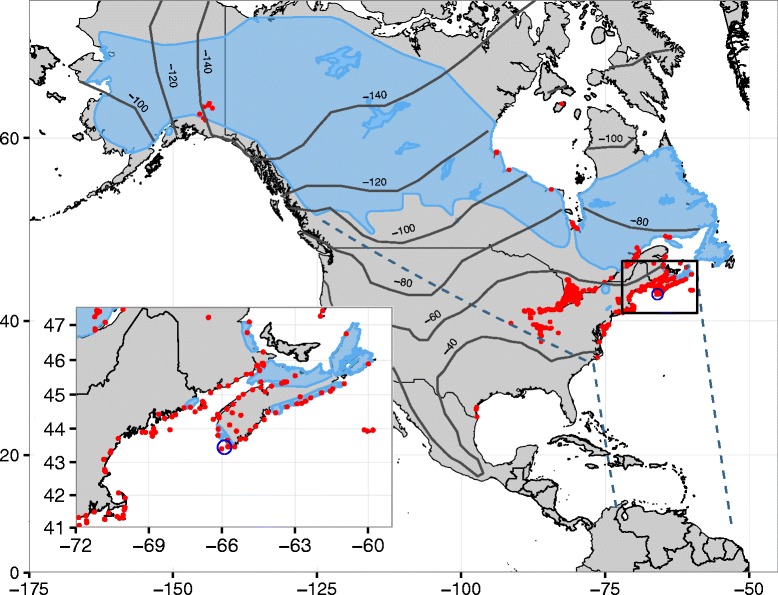
The breeding and fall migratory range for Blackpoll Warblers. The blue shaded region represents the breeding range, and the dashed lines represents their fall migratory route. The black rectangle signifies the inset region of the Gulf of Maine, and the capture locations are within the open blue circle. All automated radio telemetry towers in the Motus array during fall 2014 & fall 2015 are marked with red dots. The dark gray contours represent the isoclines derived from the isoscape in [71]. The Blackpoll Warbler breeding range was provided by BirdLife International (2014)

The Gulf of Maine extends from Nova Scotia, Canada around the Bay of Fundy to Cape Cod, Massachusetts, USA (Fig. 1). It is part of the North American Atlantic flyway. In the region, migrants from across North America, including many western and boreal breeding songbirds [39], concentrate along the Atlantic Coast. The Blackpoll Warbler (*Setophaga striata*) breeds across the boreal forests of North America from the Atlantic Coast west to Alaska [40]. During fall migration, Blackpoll Warblers from all areas of their breeding range migrate eastwards, with many stopping in the Gulf of Maine region before departing on a ~ 2500 km transatlantic flight to wintering grounds in South America (Fig. 1) [41–44]. Because of this eastward portion of their migration, Blackpoll Warblers encountered in the Gulf of Maine region vary greatly in how far they have flown from their natal areas. For example, a hatch-year Blackpoll Warbler from Alaska will have migrated ~5500 km prior to arriving in the Gulf of Maine region, while local individuals are still dispersing and exploring [34]. Hatch-year individuals from more westerly populations would likewise have experienced more route options compared to those from more eastern populations (e.g. the New England coast) as they migrated to Nova Scotia [34].

Using Blackpoll Warblers in the Gulf of Maine region as a study system, our objectives were to 1) determine the types of movement behaviours made at regional scales during migration and 2) examine how these behaviours varied with age, distance to natal origin, and capture date. We tracked individuals using the Motus telemetry system – an extensive automated radio telemetry array covering the Atlantic coast of North America (Fig. 1) [45]. Considering age as an indicator of internal state, we hypothesized that less efficient hatch-year individuals would have longer tracking durations due to lower fuel deposition rates, and would make shorter and more flights because they are less efficient flyers. We expected these differences would decrease as distance from the natal origin increased and as the migratory season progressed. Differences in navigation capacity between ages and individuals from near versus distant natal origins should result in less direct pathways and more flights oriented away from the migratory goal for hatch-years and individuals from more distant natal origins. We further predicted that hatch-year individuals would be more likely to detour around the Gulf of Maine barrier and depart from more southerly latitudes than adults, because of their reduced ability to predict weather conditions and the increased uncertainty of the distance across the barrier. Finally, we expected that individuals captured later into the season would take fewer flights, depart from locations further north, and spend less time in the region than those migrating earlier in the season. Because detections in the automated telemetry array usually occur mid-flight, we are often unable to determine true flight directions and departure locations, making analysis of how weather conditions influence flight direction and movement patterns difficult.

## Methods

### Study area and radio tagging

Migrating Blackpoll Warblers were captured in mist nets on two islands, Bon Portage Island (43°28′N, 65°45′W) and Seal Island (43°24′N, 66°0′W), situated 3 km and 24 km from the mainland of south-western Nova Scotia, Canada, respectively. Both islands are frequently used as stopping points by Blackpoll Warblers during fall migration (www.naturecounts.ca). Habitat is dominated by coniferous forest (*Abies balsamea; Picea* spp*.*) with small patches of deciduous canopy and understory (mostly *Alnus crispa* and *Amelanchier* spp.). The islands both support breeding populations of Blackpoll Warblers that depart prior to the arrival of migratory individuals of the same species [34].

Between mid-September and early November 2014 and 2015, 48 adult and 66 hatch-year Blackpoll Warblers were captured on the two islands. Individuals were banded and aged using skull ossification and moult [46]. A single, moulted retrix (usually R2 to limit effects on flight) was removed for stable isotope analysis.

### Automated digital telemetry

To track regional scale movements, individuals were fitted with a coded radio tag (Model NTQB-2, Lotek Wireless, Newmarket, Ontario) using a figure-eight leg-loop harness made with elastic thread [47]. The mass of the radio tags did not exceed 3% of an individual’s body mass. Each tag operated on the same frequency, emitting a signal consisting of a unique pattern of 4 pulses that was used to identify the individual. The tags emitted a signal at a fixed interval (burst rate) of either 11.3 or 17.6 s.

Movements were tracked using the Motus Wildlife Tracking System (www.motus.org), a coordinated network of over 300 automated telemetry towers which provides extensive coverage of the Gulf of Maine region and Atlantic coast of the United States (Fig. 1) [45]. Towers had between 2 and 6 nine-element directional Yagi antennas, and tag pulses were recorded using either Lotek SRX6000 receivers or a sensorgnome receiver (www.sensorgnome.org). Antennas were either listened to consecutively (Lotek), or simultaneously (sensorgnome). Each receiver can detect tags up to 20 km away, although topography, weather, and vegetation can all decrease detection range. Each receiving station operates on the same frequency, allowing for 24 h/day coverage for all tagged individuals in the system. For each tag detection, tag id, tower location, a GPS time stamp, antenna number, and signal strength were recorded. Detections were considered valid if there were three detections from a tag at multiples of the tag’s burst rate (± 4 ms, with the allowed error in the burst rate increasing by 1.5 ms for each missed burst, and allowing for up to 20 consecutive missed bursts).

### Stable isotope analysis

Feathers were cleaned using a 2:1 chloroform:methanol solvent and prepared for analysis at the LSIS – AFAR Bird Wing at Western University. 350 ± 20 *μ*g subsamples of individual feathers were analysed by continuous-flow isotope-ratio mass spectrometry. The comparative equilibrium approach [48] was employed to control for isotopic, non-carbon bound hydrogen exchange with two calibrated keratin hydrogen-isotope reference materials (CBS: -197‰, KHS: -54.1‰). Within-run replicate measurements (*N* = 5) of keratin standards had a standard deviation of ±4‰. Isotope values were reported in ***δ*** notation as parts per thousand (‰) deviation from the Vienna Standard Mean Ocean Water (VSMOW)–Standard Light Antarctic Precipitation (SLAP) scale.

### Analysis

Individuals were manually tracked on the islands where they were tagged. An observer attempted to find each tagged individual at least once per day to confirm if they remained on the islands. Any individuals that died before departing capture sites were removed from analysis. For individuals not detected off the capture island, we attempted to determine the direction of departure following the methods described in [23]. We used a combination of the sequence of detections on multiple on-island towers, or patterns in signal strength on one or more Yagi antennas. For example, a strong peak in signal strength followed by a long series of detections decreasing in signal strength down a Yagi antenna indicated the bird was departing in the direction of that antenna. If the departure direction was between 75° and 215°, that individual was assumed to have departed on a trans-Atlantic migratory flight. If the departure direction could not be determined, or was not oriented correctly for a trans-Atlantic flight, we classified the movement pattern as ‘ambiguous’.

For individuals detected away their capture island, a movement path was created by connecting the sequence of towers on which that individual was detected through time. If only one tower detected a bird on a given night (this happened frequently), then the true direction of a flight, as well as the start and end locations of that flight are considered unknown. Such paths do not represent the true course of an individual, but instead are a representation of the minimum distances it travelled.

Tracking duration was calculated as the amount of time between release of a tagged individual and its final detection on a tower. Cumulative distance travelled was calculated as the sum of distances between towers for the duration of tracking. Net displacement was the distance between the tagging site and the final detection. Flight tortuosity was measured using angular concordance weighted by flight distance. We considered the direction of flight (*α*) to be the rhumb line bearing between towers with consecutive detections. That measure was used to compute angular concordance weighted by flight distance (*d*) which was calculated as $\frac{1}{\sum_{i=1}^n{d}_i}\sqrt{{\left({\sum}_{i=1}^n{d}_i \cos {\alpha}_i\right)}^2+{\left({\sum}_{i=1}^n{d}_i \sin {\alpha}_i\right)}^2}$ (derived from eq. 6.8, [49]). The weighted angular concordance takes a value of 1 when all movement bearings are parallel (straight line of travel), and 0 when the movement directions cancel out (highly tortuous movement pattern with a net displacement of 0). Models of angular concordance only included individuals who were detected on at least two towers other than the tagging area (since the angular concordance of a single flight is always one). The number of flights was calculated as the sum of nights on which an individual was detected by a tower >15 km from its last known location (this distance was chosen because towers <15 km apart could potentially simultaneously detect individuals stopping over in their vicinity). Because some movements were likely missed, these values represent minimum measures of movement.

**Fig. 2:**
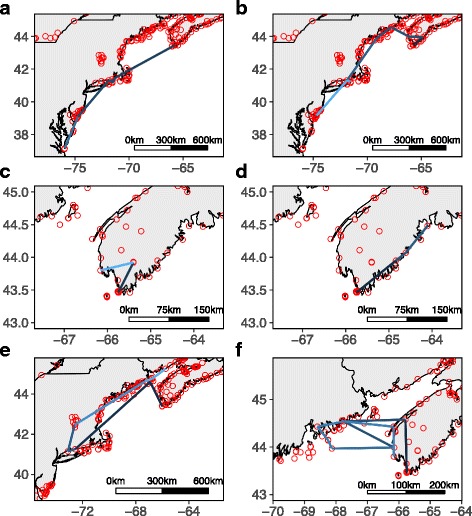
Examples of migratory (**a**, **b**) and indirect (**c** – **f**) movement patterns made by Blackpoll Warblers during fall migration. The path colour corresponds to the tracking duration, lines becoming lighter as tracking duration increases. Open red circles indicate locations of telemetry towers. **a** Migratory movement, cumulative distance = 1693 km and weighted angular concordance = 0.95. **b** Migratory movement, cumulative distance = 1392 km and weighted angular concordance = 0.75. **c** Indirect pattern, cumulative distance = 120 km and weighted angular concordance = 0.41. **d** Indirect pattern, cumulative distance = 174 km and weighted angular concordance = 0.99. **e** Indirect pattern, cumulative distance = 1576 km and weighted angular concordance = 0.13. **f** Indirect pattern, cumulative distance = 1033 km and weighted angular concordance = 0.10

An individual’s movement pattern was classified as ‘migratory’ if they were last detected below a latitude of 42°, and had a weighted angular concordance greater than 0.65. The distributions of both weighted angular concordance and net latitude were bimodal, and these numbers were the natural break points between modes. The break point for angular concordance is low enough to allow an individual to detour the Gulf of Maine (crossing at the mouth of the Bay of Fundy) and or make a short relocation flight north within Nova Scotia and still be considered migratory (Fig. 2b). For the remaining movements, there were no further break points in the distribution of distances travelled which may have indicated we were observing nested processes acting on multiple scales. As such, all other movements were analysed together as ‘indirect movement patterns’, despite high variation in the measured parameters.

The above metrics were modelled using either Gaussian (square-root-transformed total time in array, log-transformed cumulative and net displacement, and weighted angular concordance), or Gamma (number of flights), generalized linear models using a set of a priori hypotheses. The probability of an individual making a ‘migratory’ movement (includes direct departure from island) was modelled using a binomial generalized linear model.

In all models, age was included to test for effects of experience and efficiency, as were interactions between age and δ^2^H_f_ (to see if hatch-year individuals who had gained experience migrating from more distant natal origins performed differently), and age and capture date (to see if hatch-year individuals gained experience through time). δ^2^H_f_ was used to test whether natal origin influenced route selection. Capture date was included to test if movement behaviour changed as the season progressed. Individuals who departed directly from their capture site or made ‘migratory’ movement patterns were excluded from models for cumulative and net displacement, number of flights and weighted angular concordance. Since body mass and fat score were only measured at capture and would not be indicative of the condition of an individual beyond a few days, no measure of body condition was included in models of movement patterns. Year and capture site were included in all models to control for inter-annual variation and stopover-habitat effects.

Models were compared using AICc. Interaction terms were ranked low in all models so were removed from the model set. With the exception of the tracking duration models, no single model was weighted substantially more than the rest (including the null model), so we present cumulative AICc model weights and full model-averaged parameter estimates for all terms [50, 51]. The variance inflation factors (VIF) of the explanatory variables were all less than 2, and coefficients did not switch between positive and negative values, indicating low multicollinearity [52]. Parameter estimates for age, δ^2^H_f_, and capture date, as well as AICc scores and Aikake weights are provided for all candidate models in the supplementary materials [see Additional file 1]; in the text, we only discuss results for parameters with Aikake weights greater than 0.5.

We used Gaussian, identity-link generalized additive mixed models with temporally auto correlated errors (gamm, R package mgcv, [53]) with individual as a random effect, to model latitudinal and longitudinal displacement through time of individuals who did not depart directly from their capture island. In these models, interactions between time and age, δ^2^H_f_ and capture date were tested using likelihood ratio tests.

## Results

Four adult and three hatch-year individuals died on the capture island, resulting in a sample size of 44 adults and 63 hatch-years. We did not obtain δ^2^H_f_ samples from a further 3 adults and 1 hatch-year, so these individuals were removed from all models. Deuterium levels ranged from −155 to −68 and did not correlate with Julian date (*r* = 0.02, t_(102)_ = − 0.19, *p* = 0.85). Thirty-one out of 107 tagged birds were not detected away from their capture site. We determined departure directions for 11 of these individuals, 5 of which were oriented in a direction consistent with a migratory departure. The fates of the remaining 26 individuals were considered ambiguous, and they were removed from further analysis.

**Fig. 3:**
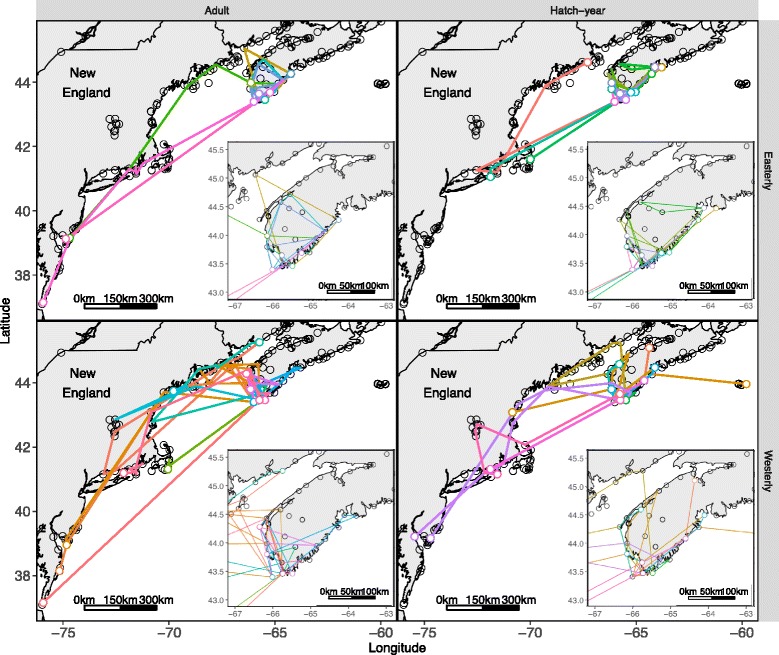
Movement paths of Blackpoll Warblers in the Gulf of Maine during fall migration. Grouped by age and feather deuterium (δ^2^H_f_). Open white circles represent last detections within the array, and paths are coloured by individual. “Easterly” δ^2^H_f_ were associated with values greater than −100

**Fig. 4:**
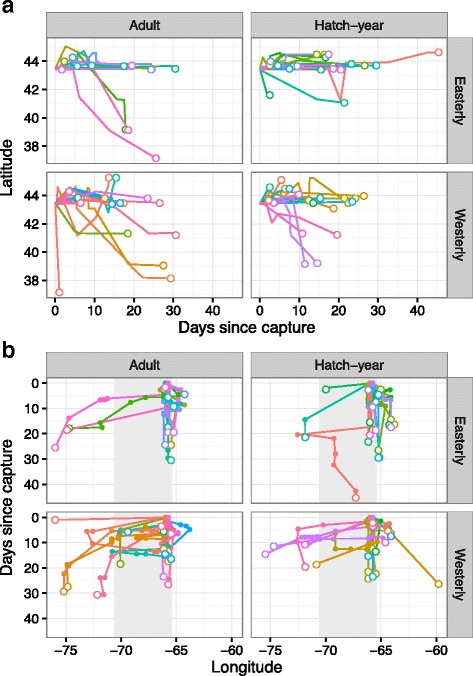
Latitudinal (**a**) and longitudinal (**b**) displacement by time of migratory Blackpoll Warblers in the Gulf of Maine. Grouped by age and feather deuterium (δ^2^H_f_). Open white circles indicate final detection, and lines are coloured by individual. On b) the approximate longitude of the Gulf of Maine is marked in gray. “Easterly” δ^2^H_f_ were considered any values greater than −100

**Fig. 5:**
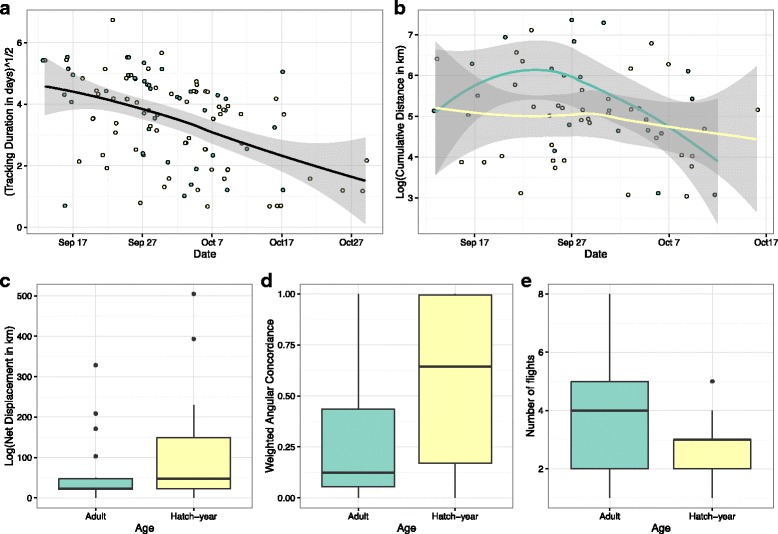
Differences in movement patterns by age, deuterium, and capture date. Movement metrics include **a** tracking duration, **b** cumulative distance, **c** net displacement, **d** weighted angular concordance and **e** number of flights. All individuals in the study who made non-ambiguous movements were included in **a**), and only individual who moved in indirect patterns are included in **b**) – **e**). Adult birds are represented by blue, and hatch-years by yellow

Of the birds who moved within the array (*n* = 76), movements were highly variable in both extent and direction (Figs. 2 and 3). Ninety-one percent of flights were nocturnal (detected between sunset and sunrise) and 80% were detected between sunset and midnight (i.e. they were not dawn reorientations [32, 54]). Latitudinal displacement through time varied with age (Fig. 4a, LRT: X^2^ = 23.4, *p* < 0.01), with adults tending to move further south through time, compared to hatch-years which remained at the same latitude. Longitudinal displacement varied by both age (Fig. 4b, LRT: X^2^ = 13.2, *p* < 0.01) and δ^2^H_f_ (LRT: X^2^ = 13.4, *p* = 0.01). Adults and individuals further from their natal origins tended to move west, while hatch-years moved anywhere from moderately east (individuals closer to natal origins) to moderately west (individuals further from natal origins). Tracking duration of all individuals combined decreased with capture date (Fig. 5a, Table 1, Table A1 [see Additional file 1]), even when the last 4 hatch-year individuals captured (after 17 October) were removed from the analysis. The model provides some support that tracking duration decreases with distance to natal origin, but the coefficients are small, and likely not biologically significant.

**Table 1:** Model averaged parameter estimates (*β* ± SE) and cumulative Akaike weights (∑ *w*_i_) for the effects of age, δ^2^H_f_, and Julian capture date on movement patterns. Generalized linear models include: the probability of making a migratory movement (binomial), tracking duration in hours, cumulative and net displacement in kilometres, and tortuosity (Gaussian), and number of flights (Gamma) of Blackpoll Warblers tracked around the Gulf of Maine during fall migration. Capture location and year were included in all models. Models of the decision to migrate and tracking durations included all individuals with non-ambiguous movements, while cumulative and net displacement, angular concordance and number of flights only included individuals moving in indirect patterns

Variable		Decision to Migrate	Tracking Time^1/2^	Log (Cum Dist.)	Log (Net Disp.)	Angular Conc.	Number of Flights
Intercept	*β* ± se	−15.6366 ± 12.4385	116.0281 ± 17.7590	4.5002 ± 2.5502	0.7596 ± 1.5955	0.0758 ± 0.9517	−0.0059 ± 0.4841
Age (Hatch-year)	*β* ± se	−0.3299 ± 0.5344	−0.3025 ± 0.8388	−0.1011 ± 0.1340	0.2010 ± 0.2165	0.2250 ± 0.1314	0.0867 ± 0.0343
	∑ w_i_	0.43	0.31	0.51	0.61	0.87	0.96
δ^2^H_f_	*β* ± se	−0.0138 ± 0.0144	0.0257 ± 0.0295	−0.0012 ± 0.0023	−0.0024 ± 0.0038	−0.0004 ± 0.0014	0.0001 ± 0.0004
	∑ w_i_	0.63	0.58	0.38	0.44	0.26	0.25
Capture date	*β* ± se	0.0475 ± 0.0434	- 0.3474 ± 0.0639	−0.0088 ± 0.0094	0.0003 ± 0.0057	0.0024 ± 0.0068	0.0010 ± 0.0018
	∑ w_i_	0.70	1.00	0.61	0.22	0.23	0.39
Capture site (Seal Island)	*β* ± se	0.4646 ± 0.5955	−0.2568 ± 1.2541	0.1784 ± 0.1295	0.2963 ± 0.1902	−0.0399 ± 0.1071	−0.0361 ± 0.0309
Year (2015)	*β* ± se	−0.2120 ± 0.6268	- 1.2884 ± 1.3381	0.1055 ± 0.1326	0.3660 ± 0.1957	0.2358 ± 0.1081	0.0552 ± 0.0333

Fourteen movement pathways were classified as ‘migratory’, however one of these individuals made a 200 km flight inland within New England (bearing of 290°), which was not consistent with the other ‘migratory’ patterns, so we classified this pattern as ambiguous and removed it from analysis. This resulted in 13 ‘migratory’ and 62 ‘indirect’ movement patterns. Combining the 5 direct migratory departures from the tagging islands with the individuals making migratory movement patterns elsewhere in the array meant that 18 individuals demonstrated direct, movement behaviour consistently oriented towards the migratory goal. The probability of making such a movement increased later in the season, and slightly with distance to natal origin (Table 1, Table A2 [see Additional file 1]). The number of regional flights recorded for migratory movements ranged between 0 and 6, with a mean ± sd of 2.3 ± 2.1 and cumulative distances for these movements (excluding direct departures) ranged from 404 to 1944 km (mean ± sd: 1139 ± 416 km).

Most individuals that moved in indirect patterns (61/62) were last detected from a location at the same latitude or further north of the capture site. Cumulative distances recorded within the array for indirect movement patterns ranged between 21 and 1576 km, with the distribution skewed towards shorter distances (median: 165 km, mean ± sd: 289 ± 348 km).

Cumulative distances travelled decreased throughout the season and were higher in adults, but hatch-years had higher net displacements than adults (Fig. 5b and c, Table 1, Tables A3 and A4 [see Additional file 1]). Null models for both cumulative distance and net displacement had AICc scores within ∆ 2 of the highest ranked models, so our confidence in these predictions are low. Individuals captured on Seal Island had higher net displacements (consistent with the increased distance of this site from the mainland), and adults and individuals from there made ~1 more flight than hatch-years and individuals captured on Bon Portage (Fig. 5d, Table 1, Table A5 [see Additional file 1]). The number of regional flights recorded for an individual ranged between 1 and 8, (mean 3.2 ± 1.5). Adults had lower angular concordance (higher path tortuosity) than hatch-years (Fig. 5e, Table 1, Table A6 [see Additional file 1]).

A total of 28 flights by 22 individuals occurred between Nova Scotia and New England with no overland detection. Flight speeds of 8 of these crossings were consistent with direct flights over the Gulf of Maine (speed >7 m/s), supporting the fact that these individuals flew between 140 and 1114 km across open water rather than detouring overland. Nine of these individuals were classified as moving in indirect patterns. Of these, 3 adults and 1 hatch-year returned to Nova Scotia, with one of these adults ultimately crossing the Gulf of Maine a total of 4 times (Fig. 2f). One hatch-year and one adult moved to southern Connecticut, before reversing and heading north to as far as northern Maine and New Brunswick (west coast of the Bay of Fundy, Fig. 2e).

## Discussion

Based on traditional views of migration, we anticipated that movements at regional scales would be highly variable, but still generally oriented towards the migratory goal [5]. Instead, only 13 out of 75 non-ambiguous movements were classified as ‘migratory’. The total amount of time spent in the region decreased, and the likelihood of making a migratory movement increased, as the season progressed, which was consistent with our initial hypothesis.

Surprisingly, many individuals moved in directions oriented away from the migratory goal (‘indirect movement pattern’). These indirect movement patterns occurred throughout the migratory season, regardless of age group and natal origin (*n* = 62). Indirect movement patterns were highly variable in both their extent and path tortuosity. We suggest that they are either an extension of ‘landscape-scale stopover movements’ [55] or a more complete representation of ‘reverse migration’, a phenomenon recorded in 10–50% of individuals each evening during migration [55–59]. Regardless, their function is unknown.

Generally, adults ranged further both latitudinally and longitudinally compared to hatch years, and adults moving in indirect patterns had lower angular concordance and made more flights than hatch-years. These results were contrary to our hypothesis that experienced adult individuals with a better underlying navigation system [26–28] would make fewer, and more direct flights. Hatch-years are more likely to make navigation errors [60], and are less selective of weather conditions during departure [12, 61] which can lead to unintentional displacements. The higher prevalence of indirect movements patterns in more experienced individuals suggests that these movements are not accidental, and thus may confer a selective advantage that has either been learned by adults, or that is too energetically costly for less efficient hatch-years. At a minimum, these movements may be an intrinsic by-product of nocternal migration that persists into adulthood [62].

Most indirect movement patterns were short (e.g. 44 individuals moved less than 250 km, Fig. 2c – d), and are perhaps best compared to landscape-scale stopover movements [55] where individuals make multiple, nocturnal relocations within their stopover landscape. Moving to inland habitats where there is lower risk of predation or better foraging opportunities is currently the most common explanation for shorter reversed movements at a barrier [56–59, 63, 64]. Many of the shorter movement patterns observed in our study still exceed the scale of landscape stopover recorded by [55], considering the median travel distance of individuals in this study was 165 km. While this is a lot of movement for a fine-scale process such as habitat selection, it seems plausible that scale of finer-scale processes (e.g. searching for appropriate foraging habitat) may be increased when an individual is in a migratory state.

A smaller proportion of individuals moving in indirect patterns made longer, regional scale flights (e.g. 11 individuals moved further than 500 km, Fig. 2e – f), that are consistent with radar observations of birds reversing over significant water barriers [54, 65]. Astonishingly, the upper range of cumulative distances flown during indirect movement patterns exceeded 1000 km, almost half the total distance that individuals must fly when they embark on their final migratory flight across the Atlantic Ocean (2540 km; [43]). We argue that these longer indirect movement patterns are so extensive they are highly unlikely to be explained by habitat preference alone. Why would an individual make several flights and fly hundreds of kilometers when suitable inland habitat can be found within 50 km of the capture site [56]?

Richardson’s radar study [54] attibuted longer reversed flights (including those across the Gulf of Maine) to a combination of late summer dispersal, dawn reorientations, and weather events. If dispersal was the cause these longer movements, we would expect hatch-year individuals from more local natal origins to have higher cumulative distances and path tortuosity compared to adults [34]. However, our results indicate either no effect, or even trends in the the opposite direction. Dawn reorientations are likewise an unlikely explaination for these movements, since 80% of flights in this study were initiated in the first half of the night. Weather may explain some of the movements we observed in this study but in that case we would have anticipated that adults would be more selective of weather conditions during departure than hatch-years [12, 61], and better able to adjust to unexpected displacements [26, 27]. In general, the effects of weather on reverse migration remain largely inconclusive, with many studies finding few effect of winds on direction of travel ([59, 62, 66], but see [57, 67]).

Ultimately, the longer, indirect movement patterns we observed do not seem well expained by any of our a-priori hypotheses, nor those in the literature. These movement patterns are counter-intuitive in front of a major barrier, where individuals would be expected to conserve and build energy reserves for the barrier crossing. Instead, individuals (including experienced adults) are allocating both time and energy towards movement that is not directly moving them towards their ultimate destination. If nothing else, we suggest that this is circumstantial evidence that flight is not the primary energetic sink during migration [68, 69].

Contrary to our hypotheses, neither age group showed an aversion to crossing the Gulf of Maine ‘barrier’, suggesting that open water crossings (at least on the scale of several 100 km) are not perceived as risky by this species during the migratory period [31, 33]. This result is consistent with other telemetry studies of warblers at relatively small water barriers during migration [33], but contrasts starkly with our observations of post-breeding Blackpoll Warblers that had bred locally in the same area [34]. During the post-breeding period, local hatch-year individuals did not cross the Gulf of Maine prior to departing across the Atlantic and adults readily crossed only at the Bay of Fundy – a much shorter distance (40 km; [34]). These large differences in movement behaviour between local and migrant individuals occurred even though local individuals left their natal islands a mere 2 weeks before the earliest migrants arrived. While the rate at which this change occurred is remarkable, it is perhaps not surprising, given that the distance across the Gulf of Maine (100–400 km) is relatively short compared to the ultimate trans-Atlantic flight that Blackpoll Warblers undertake [43].

Adults and individuals from more distant (westerly) natal origins were more likely to move south-west through time. Blackpoll Warblers with more westerly natal origins are thought to depart across the Atlantic further south compared to more easterly birds [39], so an increased inclination to move south- and west- could partially be explained by individuals correcting for an unintentional displacement north or east of their preferred migratory path. Such compensation has been shown for other species [30], including Yellow-rumped Warblers (*Setophaga coronata*) in this same location [70]. It is also possible that individuals may choose to move further south if, for example, weather or low energetic conditions make a trans-Atlantic crossing from a more northerly site riskier [31], a behaviour suggested for locally breeding adult Blackpoll Warblers at the same location [34]. Adults may be more likely than hatch-years to exhibit this behaviour since they have a more flexible navigation system [26, 27].

The high variability we observed in movement patterns, particularly indirect movement patterns, may be explained by our inability to control for movements individuals made prior to capture. Considering the prevalence of flights that we observed after tagging, we can assume that at least some individuals likely made movements within the region prior to their arrival at our capture site, which means our data under-represents the full scale of the phenomena. Furthermore, the receiving towers used in this project were concentrated around coastlines (with poorer coverage inland, especially in the north-eastern United States) which means our results are biased towards detecting movements along coasts. This, and the fact that individuals can move undetected through gaps within the telemetry array, reduces our capability of detecting the full movement pathways of all individuals.

## Conclusions

We present evidence that migrants undertake extensive flights at a regional scale during stopover – flights that are not properly accounted for in our current understanding of migration. The degree of movement observed suggests that flying is less costly than commonly assumed, or that there is an additional informational currency offsetting the costs of regional-scale flights oriented away from the migratory goal. The increased prevalence of these movements in adults suggests that they are unlikely ‘accidental’, but instead may confer an advantage that has either been learned by adults, or that is too energetically costly for less efficient hatch-year individuals. The full extent of such movements, how they vary across a broader suite of species and geographic locations, and their purpose, remain unknown. Ultimately, an individual’s decision on travel direction is determined by a combination of internal and external factors [54, 57, 64]. While explanations for reverse migration may explain a portion of the indirect movement patterns, the scope and scale of the movements that we observe here suggests that the concept of ‘reverse migration’ needs to be expanded and its causes need further exploration. Particularly, the motivations behind the longer flights observed (especially small barrier crossings), as well as individuals making multiple flights with local stopovers in-between, are not well explained by the current definitions of reverse migrations or barrier effects. It is possible that these movements are an intrinsic component of migration [62] – perhaps when individuals are in a migratory state they simply have an instinctive urge to fly.

## Additional file

Additional file 1: Table A1. Candidate models for Gaussian-distributed generalized linear models of (tracking duration)1/2 by a combination of age, δ2Hf and Julian capture date with AICc rankings and Akaike weights (wi) used for model averaging. Capture location and year (not shown) were included in all models. **Table A2** Candidate models for binomial-distributed generalized linear models of the probability of making a migratory movement by a combination of age, δ2Hf and Julian capture date with AICc rankings and Akaike weights (wi) used for model averaging. Capture location and year (not shown) were included in all models. **Table A3** Candidate models for Gaussian-distributed generalized linear models of log(cumulative displacement) for individuals moving in indirect patterns by a selection of age, deuterium value and capture date with AICc score and Akaike weight (wi) used for model averaging. Capture location and year (not shown) were included in all models. **Table A4** Candidate Gaussian-distributed generalized linear models of log(net displacement) for individuals moving in indirect patterns by a combination of age, δ2Hf and Julian capture date with AICc rankings and Akaike weights (wi) used for model averaging. Capture location and year (not shown) were included in all models. **Table A5** Candidate gamma-distributed generalized linear models of number of flights for individuals moving in indirect patterns by a combination of age, δ2Hf and Julian capture date with AICc rankings and Akaike weights (wi) used for model averaging. Capture location and year (not shown) were included in all models. **Table A6** Candidate Gaussian-distributed generalized linear models of weighted angular concordance for individuals moving in indirect patterns by a combination of age, δ2Hf and Julian capture date with AICc rankings and Akaike weights (wi) used for model averaging. Capture location and year (not shown) were included in all models. (DOCX 122 kb)
