# Supplementary material for: Migratory blackpoll warblers (Setophaga striata) make regional-scale movements that are not oriented toward their migratory goal during fall
Source: Mov Ecol. 2017 Jul 3;5:15. doi: 10.1186/s40462-017-0106-0 (PMC5494792; doi:10.1186/s40462-017-0106-0)
Supplement: Additional file 1: Table A1. — Candidate models for Gaussian-distributed generalized linear models of (tracking duration)1/2 by a combination of age, δ2Hf and Julian capture date with AICc rankings and Akaike weights (wi) used for model averaging. Capture location and year (not shown) were included in all models. Table A2 Candidate models for binomial-distributed generalized linear models of the probability of making a migratory movement by a combination of age, δ2Hf and Julian capture date with AICc rankings and Akaike weights (wi) used for model averaging. Capture location and year (not shown) were included in all models. Table A3 Candidate models for Gaussian-distributed generalized linear models of log(cumulative displacement) for individuals moving in indirect patterns by a selection of age, deuterium value and capture date with AICc score and Akaike weight (wi) used for model averaging. Capture location and year (not shown) were included in all models. Table A4 Candidate Gaussian-distributed generalized linear models of log(net displacement) for individuals moving in indirect patterns by a combination of age, δ2Hf and Julian capture date with AICc rankings and Akaike weights (wi) used for model averaging. Capture location and year (not shown) were included in all models. Table A5 Candidate gamma-distributed generalized linear models of number of flights for individuals moving in indirect patterns by a combination of age, δ2Hf and Julian capture date with AICc rankings and Akaike weights (wi) used for model averaging. Capture location and year (not shown) were included in all models. Table A6 Candidate Gaussian-distributed generalized linear models of weighted angular concordance for individuals moving in indirect patterns by a combination of age, δ2Hf and Julian capture date with AICc rankings and Akaike weights (wi) used for model averaging. Capture location and year (not shown) were included in all models. (DOCX 122 kb) [file 40462_2017_106_MOESM1_ESM.docx]

**Additional file 1: Supplementary Material to ‘Migratory Blackpoll Warblers (Setophaga striata) make regional-scale movements that are not oriented toward their migratory goal during fall’**

**Table A1** Candidate models for Gaussian-distributed generalized linear models of (tracking duration)^1/2^ by a combination of age, δ^2^H_f_ and Julian capture date with AICc rankings and Akaike weights (*w_i_*) used for model averaging. Capture location and year (not shown) were included in all models.

| **Age (hatch-year)** | **Deuterium** | **Capture Date** | **AICc** | **Akaike weight** |
| --- | --- | --- | --- | --- |
| . | 0.0443 | -0.3492 | 0.00 | 0.408 |
| . | . | -0.3502 | 0.71 | 0.286 |
| -0.9618 | 0.0436 | -0.3421 | 1.69 | 0.175 |
| -1.0292 | . | -0.3426 | 2.29 | 0.130 |
| . | 0.0456 | . | 25.58 | 0.000 |
| . | . | . | 25.78 | 0.000 |
| -1.9442 | 0.0442 | . | 25.87 | 0.000 |
| -2.0139 | . | . | 25.94 | 0.000 |

**Table A2** Candidate models for binomial-distributed generalized linear models of the probability of making a migratory movement by a combination of age, δ^2^H_f_ and Julian capture date with AICc rankings and Akaike weights (*w_i_*) used for model averaging. Capture location and year (not shown) were included in all models.

| **Age (hatch-year)** | **Deuterium** | **Capture Date** | **AICc** | **Akaike weight** |
| --- | --- | --- | --- | --- |
| . | -0.0226 | 0.0698 | 87.4 | 0.252 |
| -0.8093 | -0.0237 | 0.0756 | 87.8 | 0.206 |
| . | . | 0.0586 | 88.7 | 0.132 |
| -0.7638 | . | 0.0618 | 89.2 | 0.106 |
| . | -0.0188 | . | 89.3 | 0.102 |
| . | . | . | 89.8 | 0.079 |
| -0.6657 | -0.0188 | . | 90.1 | 0.066 |
| -0.6770 | . | . | 90.5 | 0.056 |

**Table A3** Candidate models for Gaussian-distributed generalized linear models of log(cumulative displacement) for individuals moving in indirect patterns by a selection of age, deuterium value and capture date with AICc score and Akaike weight (*w_i_*) used for model averaging. Capture location and year (not shown) were included in all models.

| **Age (hatch-year)** | **Deuterium** | **Capture Date** | **AICc** | **Akaike weight** |
| --- | --- | --- | --- | --- |
| -0.2023 | . | -0.0142 | 86.1 | 0.201 |
| . | . | -0.0142 | 86.4 | 0.174 |
| -0.2032 | . | . | 87.0 | 0.130 |
| . | -0.0035 | -0.145 | 0.97 | 0.124 |
| . | . | . | 1.13 | 0.114 |
| -0.1866 | -0.0031 | -0.0144 | 1.16 | 0.113 |
| . | -0.0034 | . | 1.96 | 0.076 |
| -0.1883 | -0.0030 | . | 2.14 | 0.069 |

**Table A4** Candidate Gaussian-distributed generalized linear models of log(net displacement) for individuals moving in indirect patterns by a combination of age, δ^2^H_f_ and Julian capture date with AICc rankings and Akaike weights (*w_i_*) used for model averaging. Capture location and year (not shown) were included in all models.

| **Age (hatch-year)** | **Deuterium** | **Capture Date** | **AICc** | **Akaike weight** |
| --- | --- | --- | --- | --- |
| 0.3162 | . | . | 130.5 | 0.251 |
| 0.3453 | -0.0058 | . | 130.7 | 0.223 |
| . | . | . | 131.1 | 0.180 |
| . | -0.0049 | . | 131.9 | 0.120 |
| 0.3161 | . | 0.0015 | 132.9 | 0.073 |
| 0.3452 | -0.0057 | 0.0010 | 133.3 | 0.062 |
| . | . | 0.0016 | 133.5 | 0.055 |
| . | -0.0049 | 0.0012 | 134.4 | 0.035 |

**Table A5** Candidate gamma-distributed generalized linear models of number of flights for individuals moving in indirect patterns by a combination of age, δ^2^H_f_ and Julian capture date with AICc rankings and Akaike weights (*w_i_*) used for model averaging. Capture location and year (not shown) were included in all models.

| **Age (hatch-year)** | **Deuterium** | **Capture Date** | **AICc** | **Akaike weight** |
| --- | --- | --- | --- | --- |
| 0.0911 | . | . | 193.8 | 0.435 |
| 0.0901 | . | 0.0025 | 194.7 | 0.286 |
| 0.0891 | 0.0004 | . | 196.0 | 0.148 |
| 0.0881 | 0.0004 | 0.0025 | 196.9 | 0.093 |
| . | . | . | 200.4 | 0.016 |
| . | . | 0.0026 | 201.3 | 0.010 |
| . | 0.0006 | . | 202.1 | 0.007 |
| . | 0.0006 | 0.0027 | 203.0 | 0.004 |

**Table A6** Candidate Gaussian-distributed generalized linear models of weighted angular concordance for individuals moving in indirect patterns by a combination of age, δ^2^H_f_ and Julian capture date with AICc rankings and Akaike weights (*w_i_*) used for model averaging. Capture location and year (not shown) were included in all models.

| **Age (hatch-year)** | **Deuterium** | **Capture Date** | **AICc** | **Akaike weight** |
| --- | --- | --- | --- | --- |
| 0.2565 | . | . | 61.7 | 0.488 |
| 0.2654 | -0.0015 | . | 63.7 | 0.178 |
| 0.2570 | . | 0.0024 | 64.1 | 0.150 |
| . | . | . | 65.5 | 0.076 |
| 0.2659 | -0.0015 | 0.0024 | 66.2 | 0.052 |
| . | -0.0008 | . | 67.7 | 0.024 |
| . | . | 0.0023 | 67.8 | 0.024 |
| . | -0.0008 | 0.0023 | 8.40 | 0.007 |
